# “Revision of subtrochanteric femoral nonunions after intramedullary nailing with dynamic condylar screw”

**DOI:** 10.1186/s12891-018-2372-4

**Published:** 2018-12-21

**Authors:** Sebastian Lotzien, Valentin Rausch, Thomas Armin Schildhauer, Jan Gessmann

**Affiliations:** 1 BG University Hospital Bergmannsheil, Bochum, Germany; 2 Department of General and Trauma Surgery, Ruhr University Bochum, Bürkle-de-la-Camp-Platz 1, 44789 Bochum, Germany

**Keywords:** Subtrochanteric nonunion, Pseudarthrosis, DCS, Dynamic condylar screw, Intramedullary nailing, Hardware failure

## Abstract

**Background:**

Nonunions of the subtrochanteric region of the femur after previous intramedullary nailing can be difficult to address. Implant failure and bone defects around the implant significantly complicate the therapy, and complex surgical procedures with implant removal, extensive debridement of the nonunion site, bone grafting and reosteosynthesis usually become necessary. The purpose of this study was to evaluate the records of a series of patients with subtrochanteric femoral nonunions who were treated with dynamic condylar screws (DCS) regarding their healing rate, subsequent revision surgeries and implant-related complications.

**Methods:**

We conducted a retrospective chart review of patients with aseptic femoral subtrochanteric nonunions after failed intramedullary nailing. Nonunion treatment consisted of nail removal, debridement of the nonunion, and restoration of the neck shaft angle (CCD), followed by DCS plating. Supplemental bone grafting was performed in all atrophic nonunions. All patients were followed for at least six months after DCS plating.

**Results:**

Between 2002 and 2017, we identified 40 patients with a mean age of 65.4 years (range 34–91 years) who met the inclusion criteria. At a mean follow-up period of 26.3 months (range 6–173), 37 of the 40 (92.5%) nonunions healed successfully (secondary procedures included). The mean healing time of the 37 patients was 11.63 months (± 12.4 months). A total of 13 of the 40 (32.5%) patients needed a secondary revision surgery; one patient had a persistent nonunion, nine patients had persistent nonunions leading to hardware failure, two patients had deep infections requiring revision surgery, and one patient had a peri-implant fracture due to low-energy trauma four days after the index surgery.

**Conclusions:**

The results indicate that revision surgery of subtrochanteric femoral nonunions after intramedullary nailing with dynamic condylar screws is a reliable treatment option overall. However, secondary revision surgery may be indicated before final healing of the nonunion.

## Background

Subtrochanteric femoral fractures account for approximately 25% of all hip fractures and have a bimodal age and sex distribution [1]. The subtrochanteric region is defined as the area between the lesser trochanter and the femoral isthmus, which is five centimeters below the trochanter [2, 3]. The femur is exposed to biomechanical forces due to the osseous anatomic conditions in the region, as well as the skeletal muscles surrounding the hip joint. Shear forces specifically affect the cancellous bone of the proximal femur, whereas bending forces especially affect the cortical bones of the subtrochanteric area and the shaft [4–6]. Nonoperative treatment of subtrochanteric femur fractures is associated with a high rate of complications [7]. Although the most recent literature does not emphasise the superiority of intramedullary fixation with nail systems (IMN), it should be however considered as a primary treatment option more particular in elderly patients [8, 9]. Nevertheless, extramedullary fixation with a 95° angled blade plate and plate-sliding screw systems also has significant value in current surgical therapy [1]. IMN seems to have biomechanical advantages due to a shorter lever arm compared to the extramedullary components. However, these advantages are partially outweighed by a difficult closed reduction [10]; screw cut outs, screw migration peri-implantary femoral fractures, malunion and nonunion are not uncommon [11–13]. General risk factors [14], as well as local risk factors due to the magnitude of trauma, can lead to delayed bone healing or nonunion in as many as 20% of subtrochanteric femur fractures [15–18]. A significant increase in nonunions of the proximal femur is expected in the future, especially in patients older than 80 years. This is due to demographic changes and the consequently greater number of proximal fractures of the femur in the population [19–21]. Nonunions of the subtrochanteric region of the femur after prior IMN can be difficult to address. Implant failure and bone defects around the implant significantly complicate the therapy, and complex surgical procedures with implant removal, extensive debridement of the nonunion site, bone grafting and reosteosynthesis usually become necessary [22]. The Dynamic Condylar Screw (DCS; Synthes, Bettlach, Switzerland) has been designed for the internal fixation of fractures of the distal and subtrochanteric regions of the femur and has superior biomechanical properties compared to the blade plate [23–25]. The purpose of this study was to evaluate a series of DCS treatments of patients with subtrochanteric femoral nonunions following IMN regarding healing rate, secondary surgeries and implant-related complications.

## Methods

**Fig. 1:**
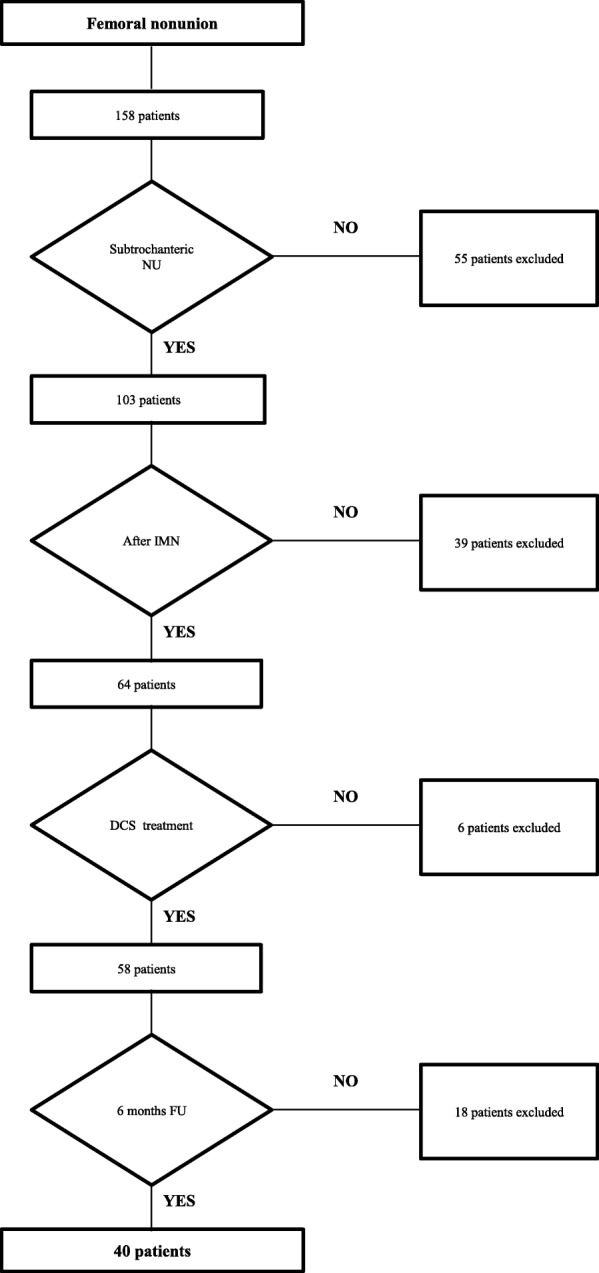
Inclusion criteria

**Fig. 2:**
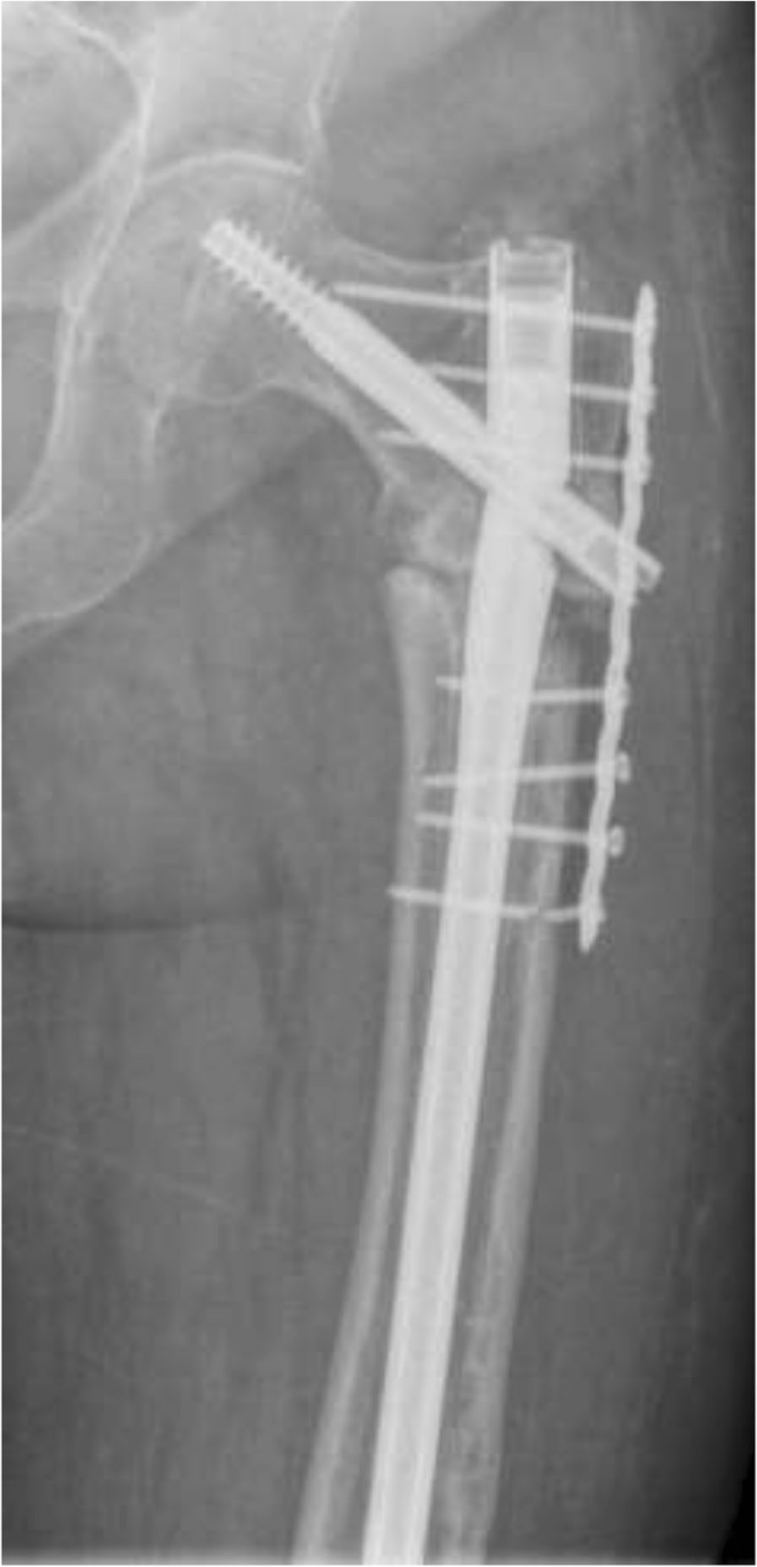
An 86-year-old female patient with a persistent femoral nonunion after nailing and having already undergone revision with augmentation plating. There is a lack of callus formation and radiographic breakage of the nail and two screws with varus deformation of the femur with a CCD of 104

The electronic medical database at the authors’ institution was searched for patients 18 years of age or older with aseptic subtrochanteric femoral nonunions after failed IMN and DCS treatment at our institution (Fig. 1). Nonunion was defined as a lack of union six months after trauma, including predictable nonunion with a lack of callus formation, gap distraction and implant breakage or progressive loosening of the nail/locking screws (Fig. 2) [26]. All patients treated with a DCS subsequent to IM nail removal (index procedure) in this time period were included in the study if an adequate follow-up period of at least six months was available. The nonunions were radiologically classified according to the pattern of callus formation. Atrophic nonunions showed little callus formation, and hypertrophic nonunions showed an extuberant callus formation with a horse-shoe or elephant-foot configuration [27]. Patients were followed through regular visits at our outpatient clinic six weeks, twelve weeks and six months after the index procedure. After the initial visits, additional visits were scheduled at different intervals until bone union was radiologically confirmed. Clinical and radiological results were analyzed at the latest follow-up examination. The primary outcome measure was time to healing calculated in months. The secondary outcome measures included complications after the index operation and hip function at the last follow-up visit. Complications were recorded regarding persistent nonunion with or without hardware failure, infection and revision surgery. Radiological results were assessed using antero-posterior and lateral radiographs. Whenever in doubt, a computerized tomography (CT) was used to confirm bony union. Union was defined as a radiologically detectable callus bridge or at least three healed cortices on the radiographs. Deep infection was defined as an infection with microbial detection leading to revision surgery [28]. Infection leading to nonunion was listed as a complication and was also counted as nonunion.

The 40 included patients had been referred to our hospital for nonunion treatment. Additional information about each patient was gathered, including demographics, comorbidities and trauma mechanism. The fractures were classified according to the Seinsheimer classification and the Orthopaedic Trauma Association (OTA) classification system as well [29]. To conduct subgroup comparisons, the patients were sorted into two different groups (Table 1). Patients without further revision surgery after the index procedure were sorted into group 1. Patients who needed secondary revision surgery were sorted into group 2.

**Table 1:** Group comparison

	Group 1 (*n* = 27)	Group 2 (*n* = 13)	*p*-value
Sex			0.58
Female	16 (59.26%)	8 (61.54%)	
Male	11 (40.74%)	5 (38.46%)	
Fracture-type^a^			0.32
Seinsheimer Type IIA	3	2	
Seinsheimer Type IIB	1	2	
Seinsheimer Type IIIA	10	3	
Seinsheimer Type IIIB	–	1	
Seinsheimer Type IV	2	–	
Seinsheimer Type V	1	–	
OTA-type^a^			0.54
32A1	1	2	
32A2	–	–	
32A3	3	2	
32B2	10	4	
32B3	2	–	
32C2	–	–	
32C3	1	–	
Trauma mechanism^b^			0.61
Low energy	16	9	
High energy	6	3	
Nonunion-type			0.53
hypertrophic	3/27 (11.1%)	2/13 (15.4%)	
atrophic	24/27 (88.9%)	11/13 (84.6%)	
Age	64.96 ± 15.87	66.31 ± 12.66	0.35
BMI^c^	26.9 ± 5.41	29.91 ± 5.6	0.57
Time to index procedure (months)	10.37 ± 7.2	8.85 ± 5.77	0.67
Prior surgeries per particpant	0.22 ± 0.42	0.69 ± 1.18	0.21
Mean operation time (minutes)	156.74 ± 42.63	149.08 ± 41.36	0.59
Number of red blood cell units tranfused	2.96 ± 3.57	3.15 ± 3.46	0.87
Lenght of hospital stay (days)	14.74 ± 5.45	15.77 ± 8.64	0.89
Diabetes mellitus	3/27 (11.1%)	0/13 (0%)	0.54
Steroid use	3/27 (11.1%)	0/13 (0%)	0.54
Smoking	3/27 (11.1%)	3/13 (23.1%)	0.37
Osteoporosis	2/27(7.4%)	3/13(23.1%)	0.31
Bony healing	27/27(100%)	10/13(76.92%)	0.03
Healing time (months)	7.96 ± 6.53	23.11 ± 26.57	0.01
Follow-up (months)	21.48 ± 17.52	36.15 ± 50.7	0.54

^a^incomplete dataset; in 15 participants Information could not be gathered due to incomplete medical records at admission to our clinic

^b^incomplete dataset; in six participants Information could not be gathered due to incomplete medical records at admission to our clinic

^c^incomplete dataset; in one participant Information could not be gathered due to incomplete medical records at admission to our clinic

### Surgical technique

**Fig. 3:**
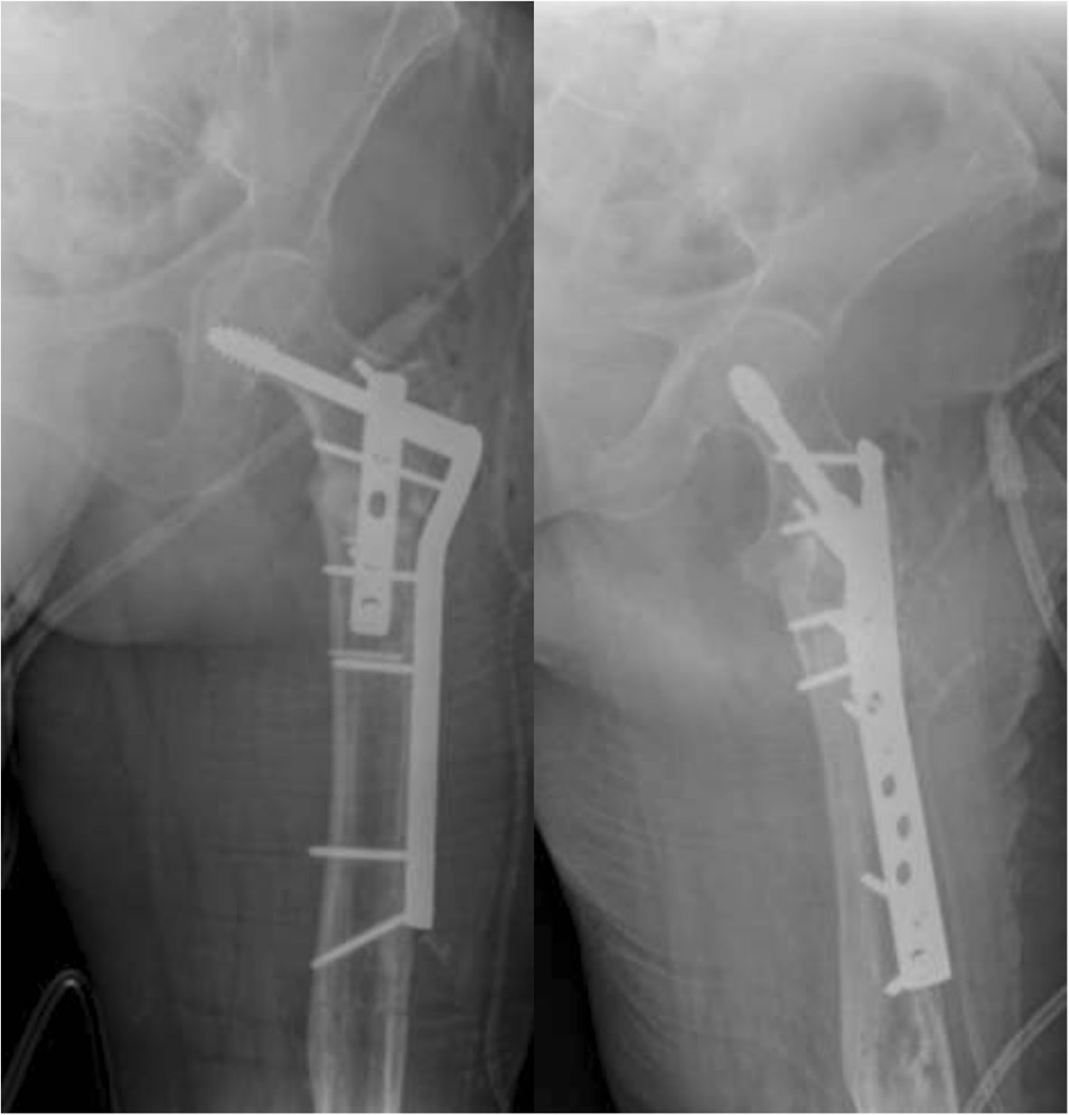
Postoperative X-rays after nail removal, restoration of the CCD (126°) and DCS treatment with a precontoured plate by leaving the augmentation plate in situ

**Fig. 4:**
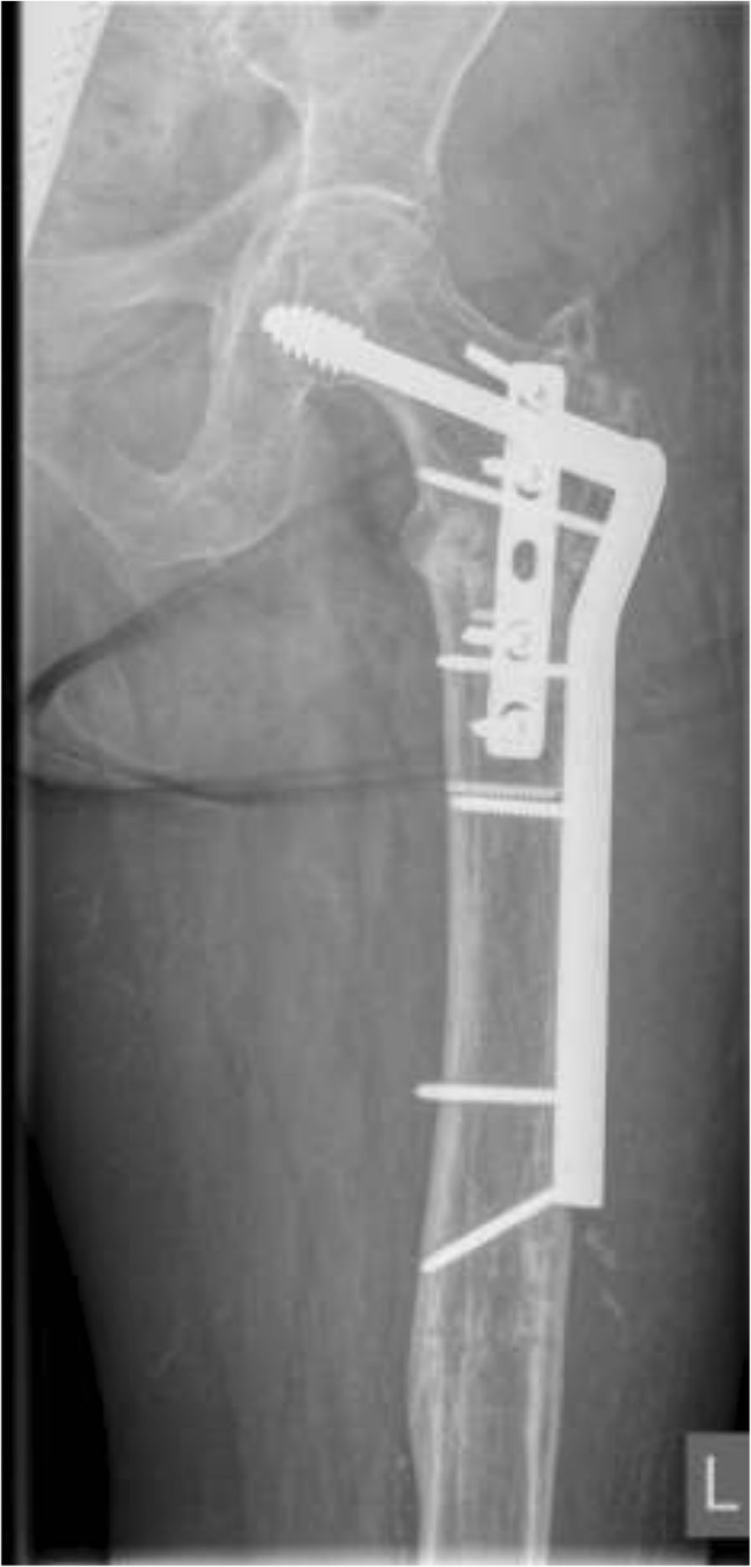
Final follow-up visit six months after index procedure showing bony union

**Fig. 5:**
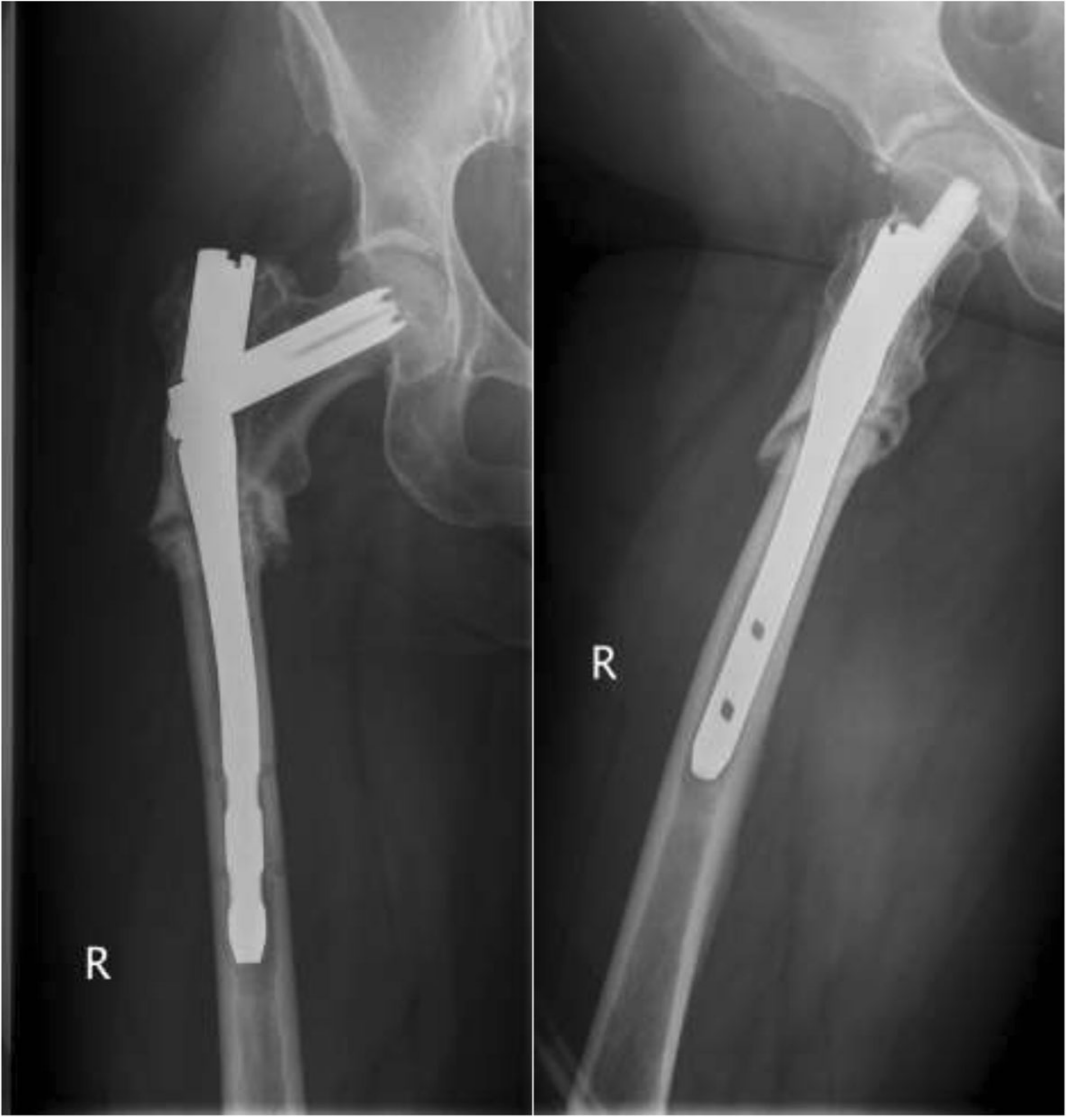
AP and lateral radiographs of a 56-year-old female patient with a hypertrophic nonunion after nailing with an implant failure and varus nonunion of the femur with a CCD of 109

**Fig. 6:**
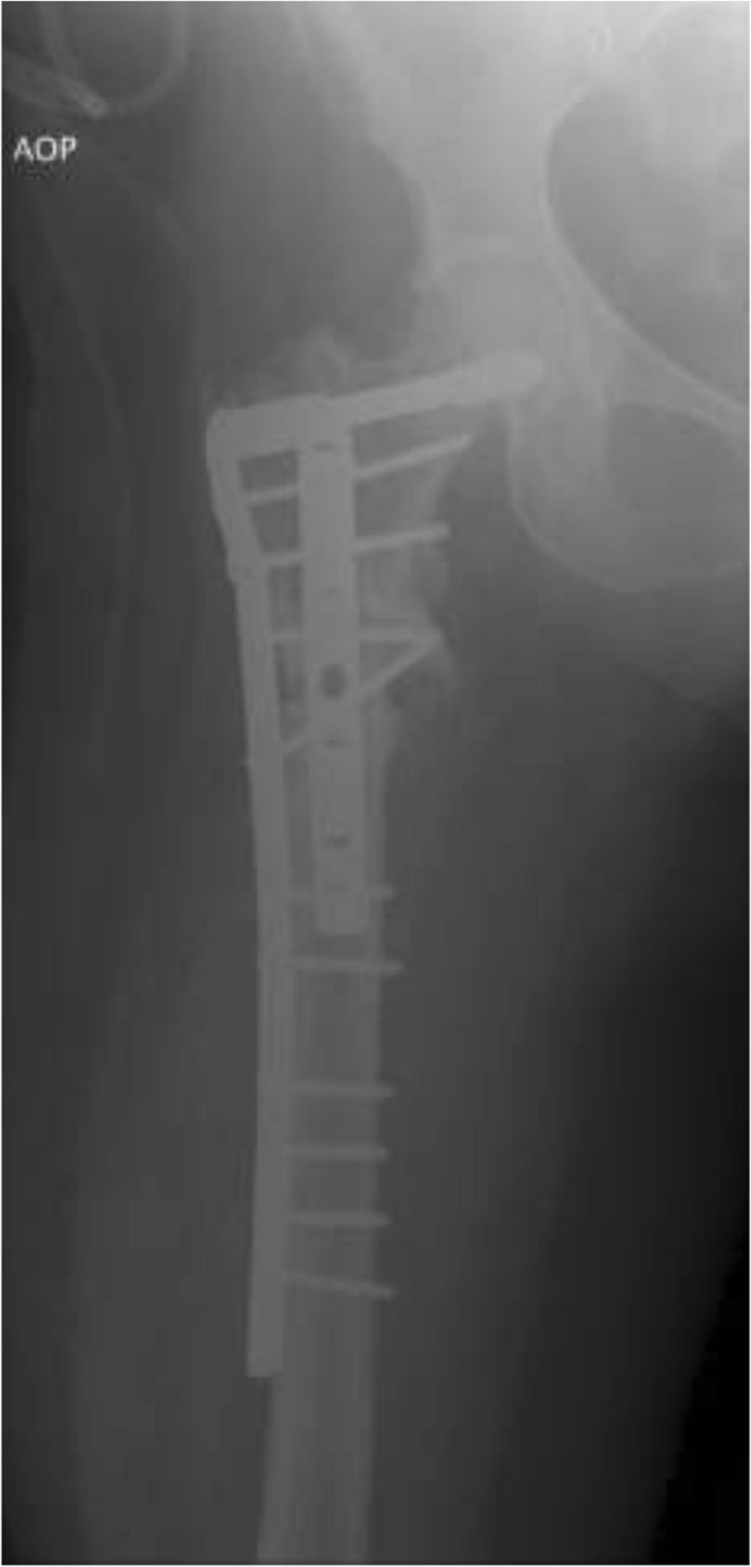
X-ray after implant removal, nonunion debridement, restoration of the CCD (131°) and ORIF utilizing a DCS with an additional LCDCP

**Fig. 7:**
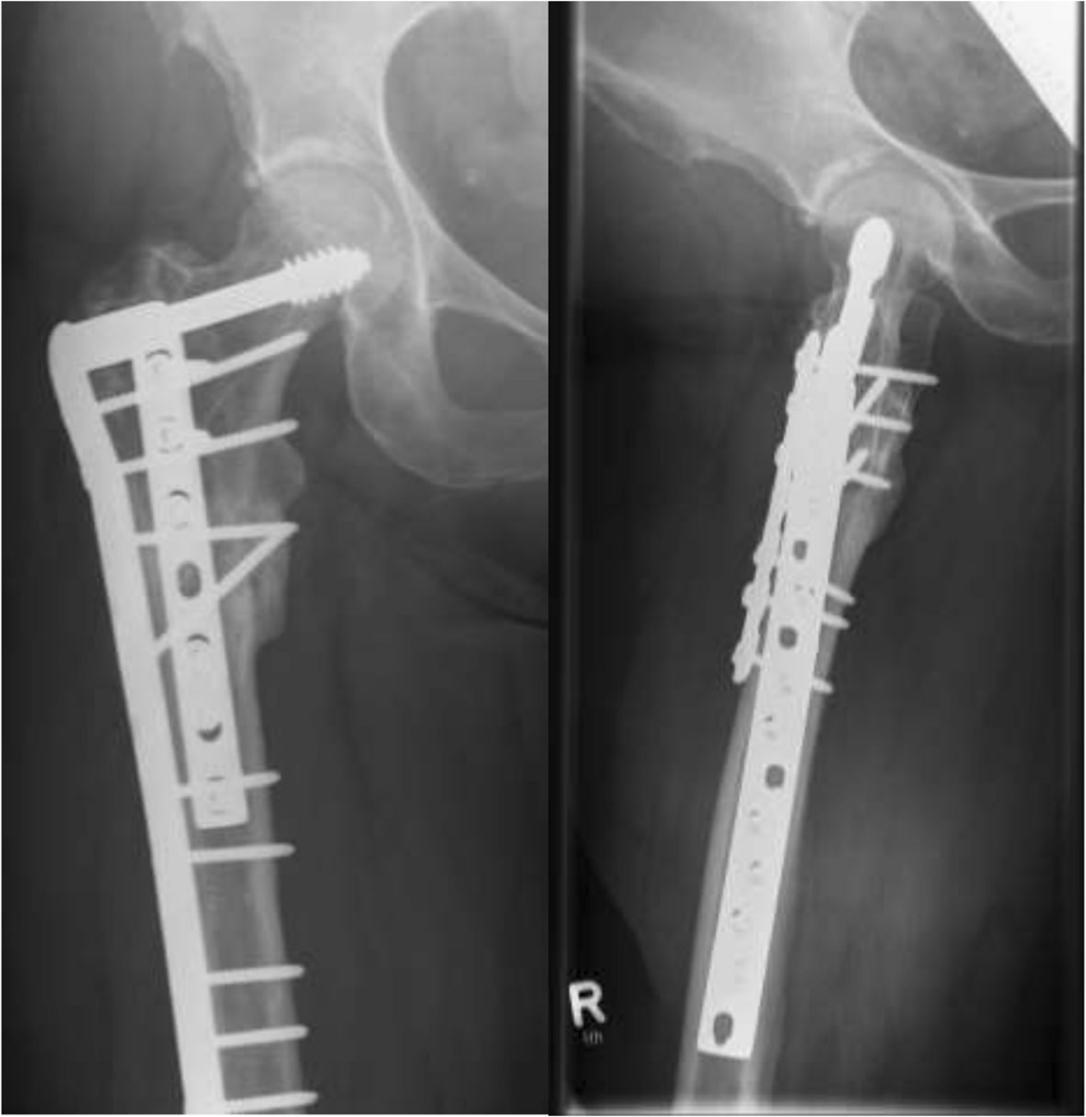
X-rays of the same patient showing a healed nonunion six months after index treatment

Eighteen different surgeons performed the index procedures. Cefazolin (two grams intravenously) was administered for perioperative antibiotic prophylaxis 30 min before the skin incision. The patient was placed in a supine position on a radiolucent table. Radiographic examinations were used to guide the osteotomy and the positioning of the implant. The initial approach was incorporated and extended distally into the lateral approach, with the fracture site fully exposed. The nail was removed and extensive debridement of the fracture site and nonunion site, including removing fibrotic tissue and necrotic bone, was performed until the bleeding bone was exposed. A collection of tissue samples was collected for microbiological analysis. After the screw insertion into the femoral neck, the DCS plate was then inserted over the lag screw. The plate length was selected according to the surgeon’s preference (9–22 holes). The CCD angle was restored by reduction of the shaft to the plate and was maintained with a reduction clamp. The degree of valgus correction depended on the preoperative measured neck shaft angle. To allow proper valgus correction, the plate may have been precontoured using plate benders before plate application (Figs. 3 and 4). An articulated tension device was used to produce compression on the nonunion zone before the additional screws were inserted. If available, cancellous bone graft harvested from the ipsilateral iliac crest was used as graft material in case of an atrophic nonunion. If not available, allograft (Tutogen Medical GmbH, Germany) was utilized. The use of an additional 4.5-mm limited contact dynamic compression plate (LCDCP) placed orthogonally onto the anterior proximal face of the femur was used at the surgeon’s discretion (Figs. 5, 6 and 7). Physical therapy was initiated one day after the index procedure. During the first six weeks, weight bearing was delayed, with toe-touch bearing allowed.

Patient characteristics were described by their mean and standard deviation, as well as their minimum and maximum values. Normality of variables was tested with the Shapiro-Wilk test. Significance was calculated using a t-test, Wilcoxon-Mann-Whitney test, contingency tables and Fischer’s exact test. A *p*-value of 0.05 or less was considered statistically significant. In the case of missing data due to the retrospective design of the study, the specific missing information was marked and explained. If further calculation was performed with an incomplete dataset, listwise deletion was used. Data were analyzed using SPSS Statistics version 23 and Microsoft Excel version 14.5.9.

## Results

Between January 2002 and July 2017, 40 patients fulfilled the inclusion criteria and were included in the study. The average length of the follow-up period was 26.3 months (range 6–173 months), with a median of twelve months. The mean age of the 24 women and 16 men upon admittance to our clinic was 65.4 years (range 34–91 years). Regarding clinical constellations, initial fractures were treated with a gamma nail (fa. Stryker) in 17 patients, and a proximal femoral nail (PFN; fa. Zimmer) was used in eight patients. Fifteen fractures were treated by Targon-, Sirius-, Trigen-, Vero-, Friedel or Orthofix intramedullary nails. Among these cases, ten of the 40 patients (23.3%) had already undergone revision surgery after nailing, including for dynamization of the nail, bone grafting, additional augmentation plate insertion or exchange nailing (Fig. 2). A total of three patients had undergone more than one revision surgery. Of the 40 total nonunions, nine (22.5%) were associated with implant failure at admission to our hospital (Fig. 5). The 40 aseptic nonunions were classified as atrophic in 35 (87.5%) cases and as hypertrophic in five (12.5%) patients.

The index surgery was performed at an average of 9.88 months (± 6.7 months) and a median of nine months after the initial nailing. The mean operation time was 154.25 min (range 70–265 min). In 18 patients (45%), an additional LCDCP was used. With the exception of ten patients, an autologous cancellous bone graft taken from the anterior iliac crest was used. In five patients, a demineralized bone matrix was applied instead of an autologous bone graft. The remaining five patients without additional bone grafting had hypertrophic nonunions. The preoperative CCD of the 40 patients prior to the index procedure was 118° (range 101° – 131°). The mean postoperative CCD was calculated to be 128° (range 114° – 142°) with a mean valgus correction of ten degrees. The mean length of the hospital stay was 15.08 days (range 6–40 days). On average, three units of red blood cells were transfused during the hospital stays (range 0–12 units).

Bony union was achieved in 27 of the 40 nonunions (67.5%) after the index procedure. A total of 13 of the 40 (32.5%) patients needed secondary revision surgery, including one persistent nonunion (2.5%), nine persistent nonunions leading to hardware failure (22.5%), two deep infections requiring revision (5%) and one peri-implantary fracture (2.5%) due to low-energy trauma four days after index surgery (Table 2). Within the study group, neither sex nor initial fracture type, trauma mechanism, nonunion type, age, BMI, time to index procedure, number of revisions prior to index procedure, operation time, number of transfused red blood cell units, diabetes mellitus, steroid use, smoking or osteoporosis was a significant factor for revision surgery after the index procedure (Table 1). Ultimately, 37 of the 40 (92.5%) nonunions healed (subsequent surgical procedures included). The mean healing time of the 37 patients was 11.63 months (± 12.4 months). In one case, the patient did not reach union and was treated with a total hip replacement (THR) seven months after the index procedure. Another 90-year-old female patient underwent a resection arthroplasty due to persistent hip infection. Additionally, a 72-year-old female did not reach union, with radiographs showing implant failure with varus deformity and screw loosening; nevertheless, she was fully mobilized without pain using forearm crutches and did not agree to further revision. Revision surgery was a significant factor in unsuccessful bony healing and prolonged healing time within the study period (bony healing *p* = 0.03; healing time *p* = 0.01).

**Table 2:** Additional information about the patients with implant failure after index procedure

Case number	Age	DCS Construction (holes)	Additional plate	Complication	Therapie
3	58	14	No	Infection	Surgical debridement leaving the plate in situ in addition to antibiotic therapy
4	67	16	No	Periimplant fracture four days after index procedure	Add. Plate
5	52	8	No	Persisting nonuinon without implant failure	Add. Plate + bone grafting
10	72	14	Yes	Implant failure; prox. Plate broken between hole 1/2	Re-ORIF with double plate construction + bone grafting
11	87	14	Yes	Implant failure; prox. Plate broken between hole 2/3	Re-ORIF+ bone grafting
14	69	16	Yes	Implant failure; prox. Plate broken between hole 3/4	Re-ORIF+ bone grafting
21	59	16	No	Implant failure; Plate broken between hole 7/8	Re-ORIF+ bone grafting
24	56	^a^	No	Implant failure; prox. Plate broken between hole 8/9	Re-ORIF with double plate construction + bone grafting
26	50	12	Yes	Implant failure; prox. Plate broken between hole 3/4	Re-ORIF with double plate construction + bone grafting
31	68	14	Yes	Implant failure; Screws broken distal 4 screws with implant loosenig	Re-ORIF+ bone grafting
40	75	16	No	Implant failure; Screw cut out	THR
53	91	12	No	Infection	Surgical debridement leaving the plate in situ in addition to antibiotic therapy
54	58	14	Yes	Implant failure; prox. Plate broken between hole 3/4	Re-ORIF with double plate construction

^a^Plate type remained unknown do to lacking data

Revision surgery was performed at a mean of 4.9 months after the index procedure (range 4 days – 12 months). In cases of a persistent nonunion with hardware failure (*n* = 9), removal of the plate and a revision procedure using the same implant again or a double plate construct (DCS + anterior LCDCP; *n* = 4) and iliac bone crafting was performed after a mean of 5.9 months (range 1–15 months). In one case, a patient developed a femoral head necrosis with screw cut-out and was treated with THR. In the case of a persistent nonunion without implant failure (*n* = 1), further revision with additional anterior plating using LCDCP and iliac bone crafting was performed. Due to infections (*n* = 2), local surgical debridement was performed leaving the plate in situ with subsequent antibiotic therapy. Implant removal was performed in seven patients after a mean follow-up period of 14 months (range 6–24 months) due to a planned foreign body removal or local pain sensation. Of the 13 patients, a subcategory of eight patients (20%) needed a second additional surgery. Second revisions were performed a mean of two months (range 6 days – 8 months) after the first revision. Second revision surgeries were necessary due to implant failure (*n* = 4), persisting nonunion without implant failure (*n* = 1) and a deep infection requiring surgical debridement (*n* = 3). In the case of a hardware failure, the same revision procedure with double plating was repeated. In the three patients with infections, debridement was repeated in two patients. In one case, resection arthroplasty resulting in a Girdlestone situation was performed. The nonunion without implant failure was treated with an iliac bone graft and demineralized bone matrix. A total of three of the 13 patients (7.5%) patients needed one or more additional surgeries, leading to two cases of osseous consolidation and one case of persisting nonunion.

At the final follow-up visit, a full range of motion of the hip was seen in ten of the 40 (25%) hips. The other 30 hips (75%) had a limited range of motion, with a minimum of 90° of hip flexion. A total of 15 patients (37.5%) could walk without any walking aids, nine patients (22.5%) walked with forearm crutches and eight patients (20%) walked with a walking frame. Two patients used a wheelchair (5%). One patient was bedridden (2.5%). In five patients, postoperative mobility could not be evaluated due to a lack of data.

## Discussion

The purpose of this study was to evaluate a series of DCS treatments of subtrochanteric femoral nonunions following IMN regarding the healing rate and implant-related complications. To the best of our knowledge, our patient series of 40 subtrochanteric femoral nonunions represents the largest study reporting on a standardized salvage DCS procedure after failed intramedullary nailing.

Nonunions of the subtrochanteric region of the femur are uncommon; however, they are difficult to treat. In addition to general risk factors [14], mechanobiological properties such as the stability of fracture fixation influence the cellular processes in the healing tissue [30]. Cephalomedullary interlocking nails have improved the results of subtrochanteric fractures with high union rates and have decreased the incidence of fixation failure [1, 31–33]. However, use of IMN remains technically demanding and requires proper reduction and correct positioning of the implant [34]. Malreduction in either the coronal (comminution of the medial cortex) or sagittal plane leads to prolonged time to union for subtrochanteric fractures [10, 35, 36]. Percutaneous reduction maneuvers and minimally invasive techniques can be useful in obtaining proper reduction [3]. Immediate unrestricted weight bearing appears to be a safe postoperative regime for subtrochanteric femoral fractures treated by IMN [1]. Early mobilization can not only lower mortality and morbidity rates but also impel functional recovery in patients with proximal femur fracture [37, 38]. If nonunion occurs after intramedullary nailing, options for reconstruction include dynamization of the nail, exchange nailing, bone grafting, augmentative plating, nail removal and plating or prosthetic replacement. The length of the proximal fragment, femoral deformities and defects in the femoral bone stock guide decision-making. The presence of hardware and poor bone stock from prior fixation attempts can compromise stable fixation [39]. In the treatment of subtrochanteric femoral nonunion, a variety of implants have been used with variable success. The treatment of subtrochanteric aseptic nonunions is rarely reported, and sample sizes in published studies have been small [39]. Barquet et al. [40] presented a study of 29 patients with subtrochanteric nonunions managed by the removal of previous implants (*n* = 9 after nailing; *n* = 20 after open reduction and internal fixation (ORIF) with blade plate, dynamic compression plate or DCS), open correction osteotomy if needed (*n* = 3), secondary osteosynthesis with a gamma nail (overreaming to provide biologic augmentation) and bone grafting in case of loss of bone substance (*n* = 5) [40]. Of these nonunions, 23 (88%) healed after the index procedure. Except for one case (96%), all nonunions healed in a mean of seven months. In total, three implant failures (11.5%) were detected. Severe comminution and fragment diastasis or varus alignment were mentioned as influencing factors for persisting nonunion [41]. Wu treated 21 patients with subtrochanteric nonunions after ORIF or IMN (all described patients remained unhealed after one to six previous surgical treatments) with locked nail stabilization [42]. The patient age ranged from 19 to 56, with a median of 36 years. No patients required additional surgery with a healing rate of 100% after one year. Nail dynamization alone offers a minimally invasive treatment option for patients without unacceptable bony deformity or limb shortening. Dynamization has been shown to be effective [43, 44], but its effectiveness according to subtrochanteric nonunions has not been proven yet. In a study including 19 patients, Kang et al. [22] showed that the union rate with the exchange of previous implants, in addition to the complete removal of fibrous tissue and bone grafting, was better than in those with retained hardware in the treatment of subtrochanteric nonunion (ten vs. nine) [22]. In contrast to the abovementioned techniques that rely on renailing or nail dynamization, plate revision of the subtrochanteric femoral nonunions has also been reported. Focusing on the enhancement of mechanical and biological preconditions (subsumed as the diamond concept [45]), Giannoudis et al. [46] presented a study of 14 subtrochanteric nonunions after initial IMN revised with ORIF (95 degree angle blade, *n* = 11) or IMN (Affixus® Hip Fracture nail, *n* = 3) [46]. In total, one revision surgery due to a blade plate failure was necessary. Finally, all 14 nonunions healed after an average of 6.8 months. De Vries used blade plating in 33 subtrochanteric nonunions (mean age of 53 years) and reported a healing rate of 96.9% (*n* = 32) [47]. The average time to healing was five months. According to the functional outcome of the united nonunions measured by the Merle d’Aubigne score [48], ten patients scored as excellent, 15 scored as good and seven as fair outcomes. In total, nine postoperative complications (27.3%) after the index surgery were mentioned. These complications required six revisions, including three “minor” revisions such as one postoperative hematoma that required drainage, one protruding tip of the plate for partial removal of the implant and one superficial wound infection requiring debridement. In total, three “major” complications led to revision surgery including one collapse of the femoral head, one implant failure leading to THR and one refracture after implant removal leading to a revision with IMN and ultimately leading to union.

According to the current literature, there is no strong evidence to support the use of either IMN or extramedullary devices in the revision of subtrochanteric nonunions. Despite the development of new implants and increasing knowledge of nonunion, the treatment of postoperative complications and persisting nonunion still presents a challenge. To date, no prospective randomized study has been published; therefore, level IV studies are currently the best available evidence [49]. We presented a large series of late plate fixations of subtrochanteric femoral nonunions following IMN. In our current study, the final healing rate was 92.5% (37/40). A significant number of the initial nonunions (87.5%) were classified as atrophic nonunions. Therefore, fracture healing seemed to be disturbed more by biology than by biomechanics. Internal fixation causes significant additional damage to soft tissues in settings where bone healing is already compromised by prior procedures and implants [50]. Positive results have been reported for closed reduction and biologic plating by DCS for treating subtrochanteric fractures [51, 52]. In contrast, extensive debridement of the atrophic nonunion sites with bone grafting [53, 54] is one of the key factors in treatment of a nonunion. Therefore, complete exposure and opening of the nonunion site is necessary for successful surgical nonunion treatment. Additionally, in cases where significant deformities require proper anatomic reduction (for patients with very short proximal fragments with complex deformities or large bone defects), the theoretical advantages of biological plating or exchange nailing are refuted.

Our data series has a relatively high number of postoperative complications leading to revision surgery (13/40). Rosso et al. [55] and Kulkarni et al. [52] reported a high implant failure rate (26%) in elderly patients (> 50 years) using DCS for unstable subtrochanteric and intertrochanteric femoral fracture treatment. Most of the abovementioned studies included younger patients [42, 47]. The mean age in Giannoudis’s study was 68.4 years. Except for one 63-year-old patient, all of the mentioned complications (*n* = 6) occurred in patients older than 74 years [46]. Our study includes a large number of elderly patients. The average age of patients upon admittance to our clinic was 65.6 years. In total, 85% patients were 50 years old or older. Most of the patients were older than 60 years (64.6%) or 70 years (41.7%) at admittance. Thus, age was not a significant factor for revision surgery in our study because both groups (Groups 1 and 2) seemed to belong to a high-risk group suffering from implant failure or complications. For these patients, less favorable results and high implant failure rates have been reported previously [56]. Cement-augmented techniques have been described and might be possible alternatives in fracture and implant fixation; however, no study has explored subtrochanteric femoral nonunion treatment [57, 58]. Hip arthroplasty after failed fixation of trochanteric and subtrochanteric fractures might be another alternative; however, several studies highlighted the challenge of the procedure with comparable or even higher complication rates [59, 60]. The strengths of the present study are the relatively large population compared to other studies focusing on nonunions after IMN and the use of one type of implant as the revision procedure.

We acknowledge the limitations of the study. First, only retrospective data gathered from a local electronic database without a defined follow-up protocol is presented. Second, there is a heterogeneity of the study cohort. We included all participants with subtrochanteric nonunions following IMN regardless of the type of initial trauma, type of nonunion and the number of prior revisions. Although there was no significant impact on bony healing in any of these categories, in our cohort group that might be a result of subgroup analysis with small group samples. Third, the index procedure was performed over the course of 15 years observational time by 18 different surgeons with varying levels of experience. Fourth, the 40 included patients had been referred to our hospital for nonunion treatment. Additional information about inital fracture treatment was limited and the rate of nonunion after fracture treatment was not available.

## Conclusions

This study shows that treatment of subtrochanteric nonunions is still a challenge. Despite high overall healing rates, a significant number of patients needed secondary surgical revisions, especially among elderly patients.

## Abbreviations

BMI: Body mass index
CCD: Neck shaft angle
CT: Computerized tomography
DCS: Dynamic condylar screw
IMN: Intramedullary nail
LCDCP: Limited contact dynamic compression plate
ORIF: Open reduction internal fixation
OTA: Orthopaedic Trauma Association
PFN: Proximal femoral nail
PRS: Prospective and randomized
